# Age-dependent effects of predation risk on night-time hypothermia in two wintering passerine species

**DOI:** 10.1007/s00442-018-04331-7

**Published:** 2019-01-03

**Authors:** Fredrik Andreasson, Andreas Nord, Jan-Åke Nilsson

**Affiliations:** Department of Biology, Section for Evolutionary Ecology, Lund University, Ecology Building, SE-223 62 Lund, Sweden

**Keywords:** *Cyanistes caeruleus*, Heterothermy, Thermoregulation, *Parus major*, Predation

## Abstract

Small animals that winter at northern latitudes need to maximize energy intake and minimize energy loss. Many passerine birds use night-time hypothermia to conserve energy. A potential cost of night-time hypothermia with much theoretical (but little empirical) support is increased risk of night-time predation, due to reduced vigilance and lower escape speed in hypothermic birds. This idea has never been tested in the wild. We, therefore, increased perceived predation risk in great tits (*Parus major*) and blue tits (*Cyanistes caeruleus*) roosting in nest boxes during cold winter nights to measure any resultant effect on their use of night-time hypothermia. Roosting birds of both species that experienced their first winter were less prone to use hypothermia as an energy-saving strategy at low ambient temperatures when exposed to increased perceived predation risk either via handling (great tits) or via predator scent manipulation (blue tits). However, we did not record such effects in birds that were in their second winter or beyond. Our results suggest that effects of increased predation risk are age- and temperature specific. This could be caused by age-related differences in experience and subsequent risk assessment, or by dominance-related variation in habitat quality between young and old birds. Predation risk could, through its effect on use and depth of night-time hypothermia, be important for total energy management and winter survival for resident birds at northern latitudes.

## Introduction

Animals that overwinter at northern latitudes face short days, limited foraging time and low ambient temperatures (*T*_a_). This requires a range of behaviours that reduce heat loss rate, which facilitates management of the daily energy budget in cold environments (reviewed by Blix 2016). In birds, this includes communal roosting (Du Plessis and Williams 1994; Hatchwell et al. 2009), use of sheltered roosting sites (Elkins 2004), postural adjustments (e.g., covering poorly insulated body parts, such as the eyes and the bill, with feathers and attaining a spherical roosting posture), and ptiloerection (Steen 1958; Hill et al. 1980; Hohtola et al. 1980).

Birds also improve their cold defense by morpho-physiological adaptations, e.g., seasonal/latitudinal increase in plumage and feather denseness (Broggi et al. 2011; Osváth et al. 2018) and improved thermogenic capacity from summer to winter (Swanson 2010). Night-time hypothermia (a reduction in nocturnal body temperature that exceeds regular circadian variation) is also a common strategy for reducing energy expenditure during food shortage or cold weather (for a review of avian facultative hypothermic responses, see McKechnie and Lovegrove 2002). Small passerines at northern latitudes have a body temperature (*T*_b_) of 41–43 °C during the day (e.g., Haftorn 1972; Nord et al. 2009; but see Lewden et al. 2014), but reduce *T*_b_ several degrees (Nord et al. 2009, 2011), and sometimes even below 35 °C (Haftorn 1972; Reinertsen and Haftorn 1986), at night. This may allow for energy savings of at least 10–30% (Reinertsen and Haftorn 1986; Cooper and Gessaman 2005). Reduced demand for metabolic fuel also relaxes the need for intensive foraging during the day, which may be important in a predator-avoidance perspective (e.g., Lima 1985, 1986; Kullberg et al. 1996). Hence, use of night-time hypothermia may substantially augment overwinter survival probability, even when the absolute reduction in overnight energy expenditure is modest (Brodin et al. 2017).

Despite the survival benefit associated with night-time hypothermia (Clark and Dukas 2000; Pravosudov and Lucas 2000; Welton et al. 2002; Cooper and Gessaman 2005; Brodin et al. 2017), birds seem to regulate nocturnal *T*_b_ at the highest affordable level (Nord et al. 2009; but see Chaplin 1976). For example, many birds maintain higher nocturnal *T*_b_ when body condition is higher and when *T*_a_ is milder (Reinertsen and Haftorn 1986; Dolby et al. 2004; Nord et al. 2009, 2011), and birds that are fed ad libitum often maintain nightly *T*_b_ that lie within the normal circadian variation (Körtner and Geiser 2000; Laurila and Hohtola 2005; Nord et al. 2009) of 1–2.5 °C (Reinertsen and Haftorn 1986; Prinzinger et al. 1991). These observations suggest that there could be costs associated with night-time hypothermia. From a physiological perspective, innate (Nord et al. 2013; Sköld-Chiriac et al. 2015; but not adaptive Nord et al. 2014) immune function, sleep (Deboer and Tobler 1996; Mueller et al. 2012) and memory retention (in connection with hibernation: Millesi et al. 2001) have all been implicated to suffer at lower *T*_b_. From an ecological perspective, increased predation risk is the most widely assumed cost of night-time hypothermia. This notion is largely based on inferences from theoretical models (Grubb and Pravosudov 1994; Clark and Dukas 2000; Pravosudov and Lucas 2000; Welton et al. 2002; Brodin et al. 2017) and the observation that birds in hypothermia are slower to respond to external stimuli (Haftorn 1972; Rashotte et al. 1998) and so could be less likely to escape an eventual predation attempt (Carr and Lima 2013). To our knowledge, only two studies have empirically evaluated the effect of increased predation risk on the use of night-time hypothermia in birds. Laurila and Hohtola (2005) showed that fasted (but not fed) domestic pigeons (*Columba livia*) kept in aviaries attenuated night-time hypothermia when subjected to an aerial predator (goshawk, *Accipiter gentilis*) during the day. Amo and colleagues (2011) exposed sleeping great tits (*Parus major*) to predatory olfactory cues (ferret, *Mustela putorius furo*), but did not find any effect on either *T*_b_ or resting metabolic rate, leading them to conclude that any anti-predatory strategies need to be taken in action before going to sleep.

There are no empirical data on how predation risk affects thermoregulation in birds in their natural environment. This is unfortunate given its potentially important effect on energy expenditure during the demanding winter period. Hence, we manipulated perceived predation risk in nest-box roosting great tits and blue tits (*Cyanistes caeruleus*) during winter and measured the resultant effect on nightly *T*_b_. We did this using two experimental setups. In the first experiment, we increased perceived predation risk for roosting great tits by first handling the birds and subsequently measuring core *T*_b_ throughout the night. In the second experiment, we increased perceived predation risk in roosting blue tits without handling them, by treating nest boxes used for roosting with a predatory olfactory cue, and also added two control groups, an olfactory control (acetic acid) and an odourless control cue (water). Olfactory cues have routinely been used to assess the responses to perceived predation risk in studies on passerines. In the majority of these studies, birds respond to a predatory scent by avoidance (Amo et al. 2008, 2011a, b, 2015, 2017; Mönkkönen et al. 2009; but see Godard et al. 2007; Johnson et al. 2011). For example, breeding blue tits become increasingly vigilant, i.e., delay their entry into the nest box and decrease the time spent inside the nest box during feeding when exposed to predatory olfactory cues (ferret) (Amo et al. 2008) in a largely similar manner as when exposed to visual predator cues (Amo et al. 2017).

If predation risk is an important ecological cost of rest-phase hypothermia (as suggested from theoretical findings), we predicted that birds exposed to increased perceived predation risk (either via handling or scent manipulation) would maintain higher *T*_b_ at night for increased vigilance and escape speed (Rashotte et al. 1998; Carr and Lima 2013). We further predicted that any such an effect would be most pronounced at low *T*_a_, because nocturnal *T*_b_ is often higher at milder *T*_a_ (Reinertsen and Haftorn 1986; Dolby et al. 2004; Nord et al. 2009, 2011).

## Methods

### Blue tits

We studied blue tits in a nest-box population surrounding Lake Krankesjön, 20 km east of Lund (55°42′N, 13°28′E) in southern Sweden (for more information about the study area, see Andreasson et al. 2016). The population contains ca. 500 nest boxes that are frequently used for roosting by blue tits during winter nights (n.b. sympatric great tits are prevented from entering the nest boxes due to the small [diameter: 26 mm] entrance hole). In January and February 2017, we added olfactory cues to the nest boxes during the afternoon (when birds were not yet in the nest boxes) and returned to nest boxes at night to measure the effects on *T*_b_ in the roosting birds. All nest boxes were checked for the presence of roosting blue tits on the night before the experimental day, by gently opening the nest-box lid without disturbing the birds. On the experimental day, ca 1 h before sunset (all boxes within 0.5–1.5 h before sunset), i.e., when birds had not yet started to roost, we added olfactory cues to the nest boxes. We added either: 20 drops of water (H_2_0; ‘control scent’); acetic acid (CH_3_COOH diluted in tap water to a concentration of 12%; ‘olfactory control scent’), or mink (*Mustela vison*) urine (‘predatory olfactory scent’; art. no 3758, supplier: Z-aim AB, Lycksele, Sweden), to a white microfiber cloth (5 × 5 cm), and attached it to the inside of the nest-box roof using a small pin. We then returned late in the evening (4.7–8.6 h after sunset), i.e., when the birds had settled, to measure *T*_b_ within 20 s of removing the bird from the nest box. This was achieved by inserting a 0.9 mm type K (chromel–alumel) thermocouple, connected to a handheld thermometer (Testo 925, Testo AG, Lenzkirch, Germany), 12 mm through the cloaca. *T*_b_ readings were not altered by further insertion. Three *T*_b_ measurements were taken and the mean temperature was used in all further analyses. The thermometer and thermocouple were calibrated at 35 °C, 40 °C, and 45 °C, by an accredited temperature laboratory (Nordtec AB, Göteborg, Sweden) before the start of the experiment. After *T*_b_ measurements, all birds were weighed (± 0.1 g), ringed, and tarsus- (± 0.1 mm) and wing (± 0.5 mm) lengths were measured. *T*_a_ was collected from a weather station in Lund, 20 km from the study site. In total, 96 nest boxes were used in the study (*n* = 32 in each category). Out of these, 4 nest boxes (predation: *n* = 1, control: *n *= 2, olfactory control: *n* = 1) were empty on the experimental night, i.e., the bird that roosted there the previous day had chosen another roosting site that night.

### Great tits

We studied great tits roosting in nest boxes in the forest of Vomb (55°36′N, 13°02′E), a pine plantation with a dense mixed-in deciduous understory, with ca. 300 nest boxes available, located ca. 10 km from the blue tit study area. Details of the study area are given in Nord and Nilsson (2012). Studies were performed during January and February 2015. After sunset, we searched nest boxes for roosting great tits. When a bird was found, we immediately measured *T*_b_ 12 mm through the cloaca using a type K thermocouple (36 gauge, 0.9 mm) connected to a data logger (OM-EL-GFX-DTC, OMEGA Engineering, Norwalk CT, USA). All birds were ringed and weighed, and tarsus- and wing lengths were measured before they were returned to the nest boxes. This handling procedure (range: 3–9 min) was probably perceived as a predation attempt by the roosting birds (cf. Nord et al. 2014; Nilsson and Nord 2017). Thus, the handling protocol increased the birds’ perceived risk of predation. Before the birds were put back into the nest boxes, we fixed the thermocouple in its place in the cloaca by attaching the thermocouple wire to two tail feathers with surgical tape and recorded *T*_b_ throughout the night (measurement interval: 2, 5, or 20 s depending on battery capacity). We then arrived before sunrise to remove the thermocouple and collect the data loggers. In total, 60 great tits were measured, but after excluding data from birds, where the thermocouple had been dislodged overnight (*n* = 13), and birds that left the box before the morning (*n* = 18), we obtained whole-night *T*_b_—profiles from 29 individuals. We smoothed the temperature profiles by creating rolling means (30 min) for each profile. We then extracted the minimum *T*_b_ during the night (*T*_b-min_) as a measure of the depth of night-time hypothermia, excluding the first 30 min after handling when the birds were still settling [cf Andreasson et al. 2018 (unpublished data)]. At our first visit, we also added an olfactory scent (homogenized mink scent glands; Leurres Forget’s Lures, Charette, QC, Canada) to half of the boxes (*n* = 30) and water to the other half (*n* = 30). However, the olfactory manipulation did not affect *T*_b_ (*t*_1,27_ = 0.76, *P* = 0.46), likely because our extensive handling protocol constituted a much greater predation cue than any olfactory stimuli. Hence, the scent manipulation was not considered further in the analyses.

### Statistical analyses

All analyses were performed in R v. 3.4.2 (R Core Team 2017). We analyzed blue tit *T*_b_ with a linear model and checked model assumptions by analyzing residual plots. Since some of the nest boxes are situated in pairs (spaced 10–20 m apart from each other) and we sometimes found roosting birds in both we included the order in which we made *T*_b_ measurements (i.e., which nest box/bird was measured first). First, we constructed a model with *T*_b_ as the dependent variable, order, treatment category and age as fixed factors, and date, time since sunset, body condition (scaled mass index, see Peig and Green 2009) and *T*_a_ as covariates. Date, time since sunset, body condition, and *T*_a_ have all been shown to influence nightly *T*_b_ in the previous studies in this blue tit population (Nord et al. 2009, 2011). We also included the three-way interaction between treatment, *T*_a_ and age, and the two-way interactions between treatment and time since sunset, and treatment and body condition. We ran this initial model with two different measures of *T*_a_ to determine which better explained variation in the data. We used: (1) the minimum *T*_a_ during the experimental night and (2) the minimum *T*_a_ from the previous night. The model with minimum *T*_a_ from the previous night provided the best fit (ΔAIC = 3.7; see Burnham and Anderson 2002) and was, hence, used in all subsequent analyses. Since there was a significant interaction between treatment category, *T*_a_, and age, we fitted separate models for the two age classes; birds in their first winter and birds in their second winter, or older (hereafter referred to as young and old birds, respectively).

*T*_b-min_ in great tits was analyzed in a similar model with *T*_b-min_ as the dependent variable, age as a fixed factor and date, time since sunset, body condition and *T*_a_ as covariates. However, minimum *T*_a_ during the experimental night was used instead (as it provided a better fit than minimum *T*_a_ during the previous night, ΔAIC = 7.0), and order was not included in the model (since the great tit nest boxes were not arranged in pairs). As all birds were handled, we were not able to evaluate any main effect of increased perceived predation risk on night-time hypothermia, but how this increased perceived predation risk interacted with age and *T*_a_ in its effect on *T*_b-min_.

Non-significant interactions (*P* > 0.05) were removed from all models. Pairwise post hoc comparisons between slopes were corrected for multiple comparisons (Tukey HSD) and estimated means for factors were calculated using the *lsmeans* package (Lenth 2016). All parameter estimates presented in tables and text are based on these estimated means (± SE) and all significances are two-tailed.

## Results

### Blue tits

**Fig. 1:**
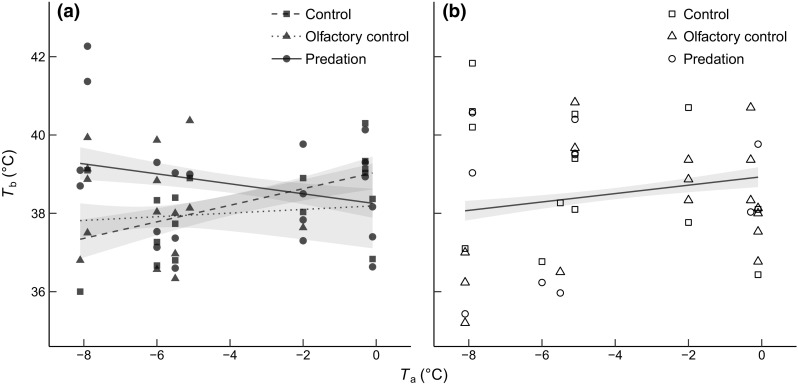
Nightly body temperature (*T*_b_) of **a** young (first winter) and **b** old (second winter or older) blue tits roosting in nest boxes treated with mink urine (predation), acetic acid (olfactory control) or water (control), as a function of minimum ambient temperature (*T*_a_) during the previous day. In **b,** the regression line is based on all old birds. Shaded bands represent model-estimated standard errors (± SE)

The relationship between *T*_a_ and *T*_b_ was different between the treatment categories in young birds but not in old birds (treatment × age × *T*_a_: *P* = 0.0033; Table 1; Fig. 1a, b). In young birds, the slope for the predatory olfactory cue (mink urine) was negative (− 0.13 ± 0.08), whereas the slopes for the control treatment (i.e., water; 0.21 ± 0.09) and the control olfactory cue (i.e., acetic acid; 0.05 ± 0.17) were both positive (treatment × *T*_a_: *P* = 0.022; Table 1; Fig. 1a). However, the slope for the predatory olfactory cue was statistically different only from the slope of the control (non-olfactory) treatment (Tukey HSD: *P* = 0.016). The slope for the control olfactory cue was not different from the slope for either the predatory olfactory cue or the control treatment (Tukey HSD: *P* ≥ 0.64). Old birds in all treatment categories (treatment × *T*_a_: *P* = 0.11) increased *T*_b_ with 0.11 (± 0.05) °C for each 1 °C increase in *T*_a_ (*P* = 0.046; Table 1; Fig. 1b), and there was no main effect of olfactory cue (*P* = 0.14; Table 1).

**Table 1:** *T*_b_ (°C) in blue tits

Variable	Estimate (SE)	*df*	*F*	*P*
All birds included				
Treatment		2, 75	0.0	0.99
Age		1, 75	0.1	0.71
*T*_a_		1, 75	4.4	**0.040**
Treatment × age		2, 75	2.0	0.14
Treatment × *T*_a_		2, 75	0.1	0.94
Age × *T*_a_		1, 75	0.4	0.51
Treatment × age × *T*_a_		2, 75	6.2	**0.0033**
Order = first	38.14 (0.14)	1, 75	11.1	**0.0014**
Order = second	38.97 (0.21)			
Date (days since January 1^st^)	0.07 (0.01)	1, 75	53.7	**< 0.0001**
Body condition (scaled mass index)	0.07 (0.13)	1, 75	0.3	0.58
Time since sunset (min)	− 0.004 (0.003)	1, 75	2.8	0.10
Young birds (first winter)				
Treatment		2, 42	1.1	0.33
*T*_a_		1, 42	0.4	0.54
Treatment × *T*_a_		2, 42	4.2	**0.022**
Mink urine × *T*_a_	− 0.13 (0.08)			
Acetic acid × *T*_a_	0.05 (0.17)			
Control (water) × *T*_a_	0.21 (0.09)			
Order		1, 42	1.4	0.24
Date (days since January 1st)	0.07 (0.01)	1, 42	24.0	**<0.0001**
Body condition (scaled mass index)	0.09 (0.17)	1, 42	0.3	0.61
Time since sunset (min)	− 0.003 (0.004)	1, 42	0.5	0.50
Old birds (second winter or older)				
Treatment		2, 31	2.1	0.14
*T*_a_	0.11 (0.05)	1, 31	4.3	**0.046**
Order = first	38.06 (0.19)	1, 31	11.8	**0.0017**
Order = second	39.25 (0.27)			
Date (days since January 1st)	0.09 (0.02)	1, 31	26.2	**<0.0001**
Body condition (scaled mass index)	− 0.004 (0.184)	1, 31	0.0	0.98
Time since sunset (min)	− 0.004 (0.004)	1, 31	0.8	0.38

For factors, estimates are least-square means (lsmeans package; Lenth 2016). For continuous variables (and their interactions with factors), estimates represent the slope of the regression between the dependent variable and the continuous variable (SE represents the fit of the regression). Significant effects (*P* < 0.05) are given in bold and effects 0.05 < *P* < 0.1 in italics

*T*_b_ was higher in birds from nest boxes that were checked last in a pair (39.0 ± 0.2 °C) compared to those that were measured first (38.1 ± 0.1 °C, *P* = 0.0014; Table 1). We also found a positive effect of date (day of the year) on *T*_b_ (*P* < 0.0001; Table 1), such that *T*_b_ increased with 0.07 (± 0.01) °C for every day into the study. Body condition (*P* = 0.58) and time since sunset (*P* = 0.10) did not influence *T*_b_ (Table 1).

### Great tits

**Fig. 2:**
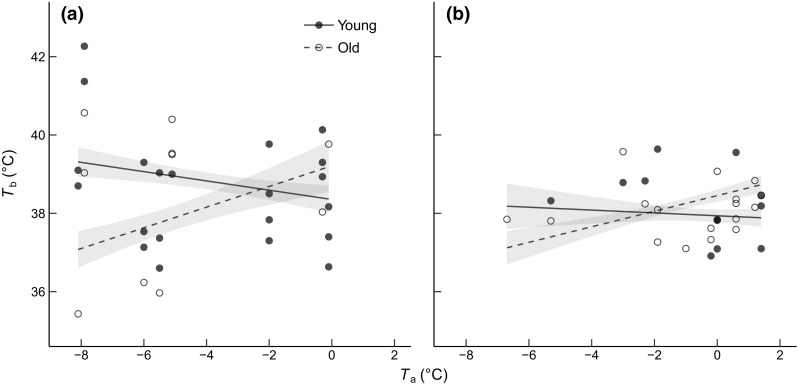
Nightly **a** body temperature (*T*_b_) in blue tits and **b** minimum body temperature (*T*_b-min_) in great tits roosting in nest boxes as a function of minimum nightly ambient temperature (*T*_a_), separated on young (first winter) and old (second winter or older) birds. All birds were exposed to an increased perceived predation risk, either via handling (great tits) or via olfactory cues (blue tits). Shaded bands represent model-estimated standard errors (± SE)

The effect of *T*_a_ on *T*_b-min_ in previously handled great tits was also different in the two age classes (*P* = 0.022; Table 2; Fig. 2b), such that *T*_a_ in old birds had a positive effect on *T*_b-min_ (slope: 0.20 ± 0.07), whereas the relationship between *T*_a_ and *T*_b-min_ in young birds was slightly negative (slope: − 0.04 ± 0.09). Both date and time since sunset influenced, or tended to influence, *T*_b-min_ negatively (*P* = 0.030 and *P* = 0.065, respectively; Table 2), whereas body condition had a positive effect on *T*_b-min_ (*P* < 0.0001; Table 2).

**Table 2:** *T*_b_ (°C) in great tits

Variable	Estimate (SE)	*df*	*F*	*P*
Age		1, 22	5.1	**0.035**
*T*_a_		1, 22	1.5	0.24
Age × *T*_a_		1, 22	6.1	**0.022**
Young × *T*_a_	− 0.04 (0.09)			
Old × *T*_a_	0.20 (0.07)			
Date (days since January 1st)	− 0.03 (0.02)	1, 22	5.4	**0.030**
Body condition (scaled mass index)	0.58 (0.10)	1, 22	30.3	**< 0.0001**
Time since sunset (min)	− 0.001 (0.001)	1, 22	3.8	*0.065*

For factors, estimates are least-square means (lsmeans package; Lenth 2016). For continuous variables (and their interactions with factors), estimates represent the slope of the regression between the dependent variable and the continuous variable (SE represents the fit of the regression). Significant effects (*P* < 0.05) are given in bold and effects 0.05 < *P* < 0.1 in italics

## Discussion

Young, but not old, blue tits exposed to an olfactory predatory cue increased night-time *T*_b_ at low *T*_a_ compared to birds manipulated with water (Figs. 1a, 2a). Great tits that were manipulated with a simulated predator attack via handling (Fig. 2b) also showed a qualitatively similar age-related effect of *T*_a_ on *T*_b_. These results are compatible with young birds prioritizing the ability to detect and/or escape a predator attack when *T*_a_ is further below the lower critical temperature. Why then, do young birds attenuate rest-phase hypothermia in response to increased predation risk at low *T*_a_, when old birds do not?

Age can influence decision-making, at least insofar that older birds have more experience and are possibly more precise in their assessment of risk, whereas younger (inexperienced) birds might adopt a “better safe than sorry”-strategy (Rodriguez-Prieto et al. 2008). It is also possible that cues from predators are quite common even in the absence of manipulation, but that actual predation attempts are rare. If so, older birds with more experience may use hypothermia at low *T*_a_, knowing that the risk of predation is small in spite of olfactory cues being present. The intermediate effect of the olfactory control treatment (acetic acid) on night-time *T*_b_ in relation with *T*_a_ suggests that it is likely that young birds recognize and detect the odour, which could be attributed to fear or aversive behaviour associated with neophobia, as previously reported in domestic chickens (Jones et al. 2002). The age-dependent effect of increased perceived predation risk on night-time hypothermia was primarily evident at low *T*_a_, indicating that potential age-related differences in experience and risk assessment are most pronounced at low *T*_a_. At milder temperatures, birds in all treatment categories and of all ages seem to maintain a *T*_b_ high enough for being able to escape a possible predation attempt.

The above explanation assumes that older birds are acting adaptively and that young birds are failing to do so, because they are less apt at making correct assessments of predation risk. An alternative explanation could be that it is the young birds that are acting adaptively and that older birds are failing to do so, possibly due to physiological “wear and tear” processes associated with aging, which could impair thermoregulatory capacity negatively. Basal metabolic rate has been shown to decrease with age in both great tits (Bouwhuis et al. 2011, cross-sectional data; Broggi et al. 2010, longitudinal data), and zebra finches (Moe et al. 2009, longitudinal data) (but see Moe et al. 2007 for no such effects in long-lived snow petrels). Whether the maximum metabolic rate birds can achieve for thermogenesis (i.e., summit metabolic rate; e.g., Swanson 2010) shows similar age-related declines is not well understood. Moreover, since only 17 of the 39 older blue tits were of a known age, we have limited knowledge of the age distribution of the remainder of the birds that were in their third calendar year or older, making any physiological “wear and tear” more difficult to detect.

Older birds are dominant (Krams et al. 2013a) and it is possible that they have prior access to nest boxes in winter territories of high quality (i.e., low predation risk and/or high food availability) and that sub-dominant, young birds have to choose nest boxes in territories of low quality. In a territory, where nocturnal predation risk is lower than that during daytime, deeper night-time hypothermia might allow birds to retain enough energy reserves to leave the nest box later in the morning, thereby minimizing predation risk from crepuscular predators. However, we were unable to find any large-scale age-dependent spatial distribution in the study area, i.e., the distribution of young and old birds did not differ between the five main sites within the study area (*χ*^2^ = 4.2; *df *= 4; *P* = 0.38). It would be highly useful in future studies to assess whether or not age-dependent spatial distribution can be influenced by predation pressure and if so, to what extent.

Alternatively, older birds could be more likely to accept a slightly higher predation risk overnight, because their residual reproductive value is lower compared to that of young birds (Clark 1994; Wolf et al. 2007). In line with this, young birds of some species seem to adopt more active predator-avoidance strategies than older conspecifics. For example, juvenile willow tits (*Poecile montanus*) are more likely to flee following conspecific alarm calls compared to adults (Rajala et al. 2003), and young blackbirds (*Turdus merula*) are also more likely to use flying as an escape route when a predator approaches, whereas adult birds typically run away from threats (Rodriguez-Prieto et al. 2008). Similar patterns have also been observed in mammals (Ramakrishnan and Coss 1999).

Young female great tits have also been shown to increase heterophil/lymphocyte (H/L) ratio during a cold spell (Krams et al. 2011), which suggests that they were experiencing elevated physiological stress during this time. Since higher H/L ratio has been showed to predict lower humoral immune response to a novel antigen (Krams et al. 2012, 2013b) and more stress-related behaviours (Krams et al. 2013b), it is possible that the young birds in our study had higher stress reactivity, which might have prevented them from decreasing their *T*_b_ at low *T*_a_.

In blue tits, the order in which the birds were measured in a nest-box pair had a strong effect on *T*_b_, with birds measured last having a *T*_b_ 0.9 °C higher than those measured first (Table 1). This would be expected if birds were able to hear our approach to the first nest box, resulting in increased alertness and vigilance in preparation for what they might perceive as a potential impending predation event. Thus, in addition to olfactory cues and handling, auditory cues also seem to play a role in assessment of predation risk and subsequent management of nightly *T*_b_.

## Conclusions

We have shown that young, but not old, blue tits maintain higher *T*_b_ at low *T*_a_ when exposed to an increase in perceived predation risk. We obtained a qualitatively similar result in the closely related great tit in response to a handling-induced increase in perceived predation risk. Our results, therefore, support the, until now, largely theoretical assumption that predation risk is a possible ecological cost of night-time hypothermia. In this sense, nocturnal predation risk could also be an important factor for the total energy budget management in small passerines that winter in cold environments. Importantly, we found evidence for age-specific effects of increased predation risk, which could be related to intrinsic differences between young and old birds (such as age-related effects on experience in risk assessment) or dominance-related variation in habitat quality between young and old birds. Thus, a complete assessment of the factors governing energy intake and expenditure in the little bird in winter (sensu Brodin 2007; Brodin et al. 2017) by necessity needs to take into account not only intrinsic variation in physiology, but also aspects relating both to the environmental quality, predator–prey interactions, and the summed experiences gained by the animal during its life.
